# The ictal wavefront is the spatiotemporal source of discharges during spontaneous human seizures

**DOI:** 10.1038/ncomms11098

**Published:** 2016-03-29

**Authors:** Elliot H. Smith, Jyun-you Liou, Tyler S. Davis, Edward M. Merricks, Spencer S. Kellis, Shennan A. Weiss, Bradley Greger, Paul A. House, Guy M. McKhann II, Robert R. Goodman, Ronald G. Emerson, Lisa M. Bateman, Andrew J. Trevelyan, Catherine A. Schevon

**Affiliations:** 1 Department of Neurological Surgery, Columbia University Medical Center, New York, New York 10032, USA; 2 Department of Physiology and Cellular Biophysics, Columbia University, New York, New York 10032, USA; 3 Department of Neurosurgery, University of Utah, Salt Lake City, Utah 84132, USA; 4 Institute of Neuroscience, Newcastle University, Newcastle upon Tyne NE1 7RU, UK; 5 California Institute of Technology, Division of Biology and Biological Engineering, Pasadena, California 91125, USA; 6 Department of Neurology, UCLA David Geffen School of Medicine, Los Angeles, California 90095, USA; 7 School of Biological and Health Systems Engineering, Arizona State University, Tempe, Arizona 85287, USA; 8 Department of Neurosurgery, Icahn School of Medicine at Mount Sinai, New York, New York 10029, USA; 9 Department of Neurology, Weill Cornell Medical College, New York, New York 10021, USA; 10 Department of Neurology, Columbia University Medical Center, New York, New York 10032, USA

## Abstract

The extensive distribution and simultaneous termination of seizures across cortical areas has led to the hypothesis that seizures are caused by large-scale coordinated networks spanning these areas. This view, however, is difficult to reconcile with most proposed mechanisms of seizure spread and termination, which operate on a cellular scale. We hypothesize that seizures evolve into self-organized structures wherein a small seizing territory projects high-intensity electrical signals over a broad cortical area. Here we investigate human seizures on both small and large electrophysiological scales. We show that the migrating edge of the seizing territory is the source of travelling waves of synaptic activity into adjacent cortical areas. As the seizure progresses, slow dynamics in induced activity from these waves indicate a weakening and eventual failure of their source. These observations support a parsimonious theory for how large-scale evolution and termination of seizures are driven from a small, migrating cortical area.

## Abstract (web summary)

Epileptic brains display inhibitory restraint as manifested by the spread of synchronized activities being delayed in timing. Here, Elliot Smith and colleagues show fast-moving traveling wave that originates from the edge of ictal wavefront with subsequent depolarization and multiunit firing in the seizing brain regions in epileptic patients.

## Graphical abstract

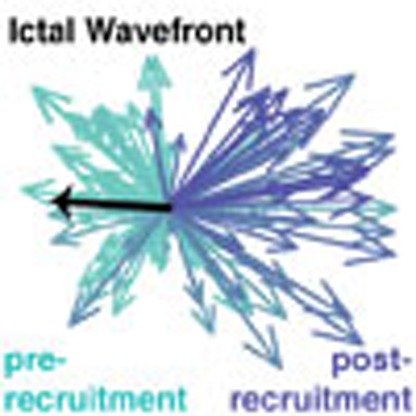

Despite over 50 years of research into seizure electrophysiology, there remains a limited understanding of the evolving spatial and temporal structure of seizures. This knowledge gap undoubtedly contributes to the enduring mystery surrounding many open questions in epilepsy research pertaining to seizure origin, evolution and termination. Clinical electroencephalography (EEG) recordings of focal seizures consistently show a progressive expansion of synchronized pathological activity to large cortical regions, followed by abrupt self-termination that occurs simultaneously across all regions^1^,^2^. The question of how the synchronized pathological activity is coordinated over expansive cortical areas remains unanswered, yet is a fundamental issue for both basic research and clinical studies that rely on the use of EEG to identify sites of seizure origin.

It is currently presumed that seizure activity in humans is generated from a cohesive cortical network spanning potentially several centimetres^2^,^3^,^4^,^5^,^6^; however, the linking mechanism for nodes in these networks is yet undetermined. Animal studies, on the other hand, have catalogued a range of spatially focused cellular or ionic changes during epileptic events^7^,^8^,^9^,^10^,^11^. The challenge is to provide a coherent explanation that encompasses the discrepancy in scale between these animal models of seizures and the clinical observations^12^.

In our previous work, we described how extensive cortical local field potentials (LFPs) can be driven from a small cortical area exhibiting strong neuronal population firing^13^,^14^. We therefore proposed a spatiotemporal structure for seizures that corresponded with observations from both animal models^15^,^16^,^17^ and spontaneous human seizures^13^,^18^. In this structure, the seizing territory is led by a slowly advancing, sharply demarcated, narrow (<2 mm) band of continuous (tonic) multiunit firing, termed the ictal wavefront^13^,^19^. Behind this wavefront, in the seizing territory, there are synchronized rhythmic discharges that give rise to the classic EEG signature of seizures over broad areas of cortex. While the ictal wavefront is difficult to identify in standard or intracranial EEG recordings, the synchronized activity in the seizing territory can be detected from high-frequency LFP^14^.

Here we conduct a detailed investigation of the spatiotemporal dynamics of the seizing territory in the human brain, to characterize mechanisms of seizure evolution and termination. We hypothesize that, as previously reported in a murine slice model^15^, fast-moving travelling waves originating from the ictal wavefront trigger intense depolarizations and associated multiunit firing in the seizing territory. That these ictal discharges arise from the ictal wavefront indicates that the ictal wavefront is the focal point for the mechanisms of seizure origin, propagation and termination. Specifically, we hypothesize that gradual reduction in the excitatory currents generated from a slowly weakening ictal wavefront is sufficient to explain the evolution of both field potentials and multiunit activity (MUA) in the seizing territory, including the seizure's eventual spontaneous termination.

## Results

### Natural epochs in spontaneous human seizures

Recordings of clinical seizures were analysed in five patients implanted with microelectrode arrays (MEAs), as previously described^13^,^20^,^21^. These were 4 × 4 mm ‘Utah-style' arrays, with 1-mm long electrodes designed to record unit activity and LFPs from adjacent tissue in neocortical layers 4 and 5, and 400 μM spacing between the electrodes^13^,^22^. In three of these patients (patients 3, 4 and 5; see Supplementary Table 2; six seizures), the MEA was located within the cortical region invaded by the seizure. Following terminology we introduced previously^13^, we term this region the ‘ictal core'. In these three patients, the MEA captured the expansion of the ictal core after seizure initiation, which was not captured directly. While all of the MEAs were implanted into the clinically defined seizure onset zone, in two patients, the seizures did not invade the sites of MEA implantation (patients 1 and 2, 10 seizures); that is, the MEAs were in ‘penumbral' sites, which remained outside the ictal core for the entire duration of each seizure (Supplementary Fig. 1). As previously reported, these penumbral areas were characterized by an absence of intense, synchronized MUA despite exhibiting low-frequency LFP that was otherwise typical of seizure activity^13^.

**Figure 1:**
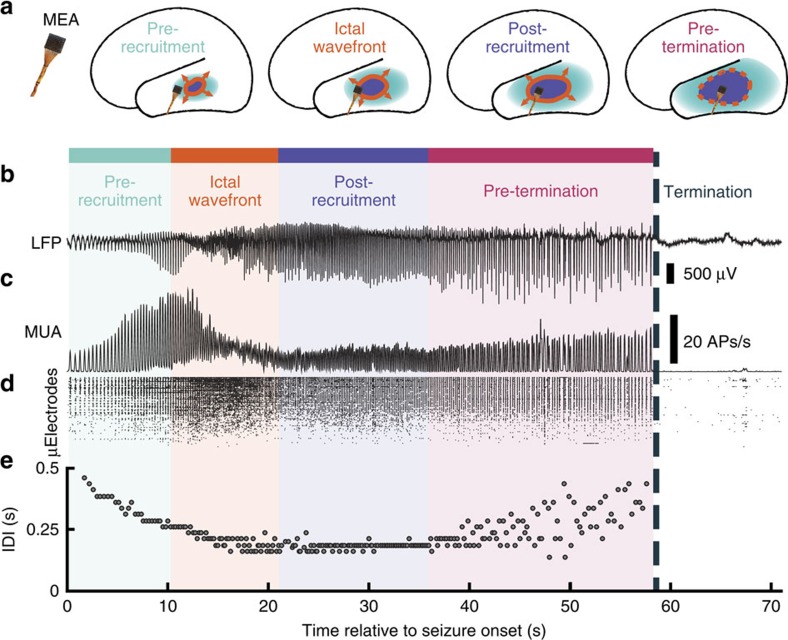
Progression of seizure activity recorded from a microelectrode array in the ictal core. Colours indicate the different epochs in the seizure: light blue is the pre-recruitment epoch, orange is the ictal wavefront epoch, purple is the post-recruitment epoch and pink is the pre-termination epoch. Seizure termination is labelled with a dark blue dotted line. (**a**) Cartoon of hypothesized spatial organization of seizure activity relative to the microelectrode array (MEA) location. (**b**) Raw LFP traces recorded from a single microelectrode during a seizure. (**c**) Averaged firing rate over electrodes on the array during the seizure in **b**. (**d**) Multiunit raster plot over MEA channels during the seizure in **b**. (**e**) Inter-discharge intervals (IDIs) for each discharge through the duration of the same seizure. The abrupt transition from regular to irregular IDIs at 36 s after seizure onset marks the transition from post-recruitment (period following passage of the ictal wavefront) to the pre-termination epoch. In both **b** and **c**, the abrupt cessation of discharges in both LFP and MUA, which is the defining event marking seizure termination, is evident.

To address our hypotheses regarding the seizure generating territory, this article focuses on the three patients in which the MEAs were recruited into the ictal core. For these patients, seizure invasion at the MEA site was identified by the abrupt appearance of transient, intense and asynchronous multiunit firing referred to as the ictal wavefront, marking the moment of failure of strong inhibitory restraint^13^,^19^,^23^. This period of intense firing was followed by a rhythmic train of ‘ictal discharges,' that is, high-amplitude field potential deflections associated with transient bursts of multiunit firing and high-γ activity, alternating with periods of relative silence between discharges (Fig. 1b,c). The dominant rhythm of ictal discharges gradually decreased in frequency towards the end of the seizure (Fig. 1d). On the basis of these observations, the MEA data from these seizures were subdivided into the following readily identifiable phases (Fig. 1a): (1) a pre-recruitment epoch, in which ictal discharges may begin to appear in the low-frequency LFP; (2) the steady multiunit firing characterizing the ictal wavefront and recruitment into the seizure; (3) a post-recruitment epoch, following passage of the ictal wavefront, in which the inter-discharge intervals (IDIs) remain stable; and (4) a pre-termination epoch (mean±s.d. duration=16.80±8.35 s) in which the pace of the ictal discharge rhythm gradually slows and becomes irregular, until the seizure abruptly terminates (Fig. 1; Supplementary Fig. 2a).

Such a transition from regular ictal discharging to slowing, irregular ictal discharges has been observed as a signature of approaching seizure termination for decades^24^. Here we quantified when this signature appeared (Methods), and operationally defined said time as the division between post-recruitment and pre-termination epochs. The range of division times was consistently small across MEA channels (s.d.=3.72 s) and electrocorticography (ECoG) channels for each seizure (s.d.=6.07 s). Furthermore, IDI distributions were not significantly different between seizures recorded on MEA and ECoG (two-sample Kolmogorov–Smirnov tests, all *P*≥0.52; Supplementary Fig. 2b). There was no significant difference in the post-recruitment/pre-termination division times determined from MEA recordings compared with those determined from ECoG (Mann–Whitney *U*-tests, both *P*≥0.3; Supplementary Fig. 2c), indicating that this phase transition occurs simultaneously in all tested channels.

### Ictal discharges form travelling waves

Previous work has shown that travelling waves of synaptic potentials propagate across the brain under normal sensory processing^25^,^26^ (reviewed in ref. 27). Travelling waves have also been demonstrated by assessing site-to-site delays in high-amplitude peaks of epileptiform discharges^28^,^29^,^30^. Travelling waves appeared in this study during ictal discharges: high-amplitude LFP events with duration up to 200 ms, moving rapidly across the cortical surface. In contrast, the slowly propagating ictal wavefront is distinguished from travelling waves by its composition of steady firing lasting several seconds with minimal effect on low-frequency activity^13^.

**Figure 2:**
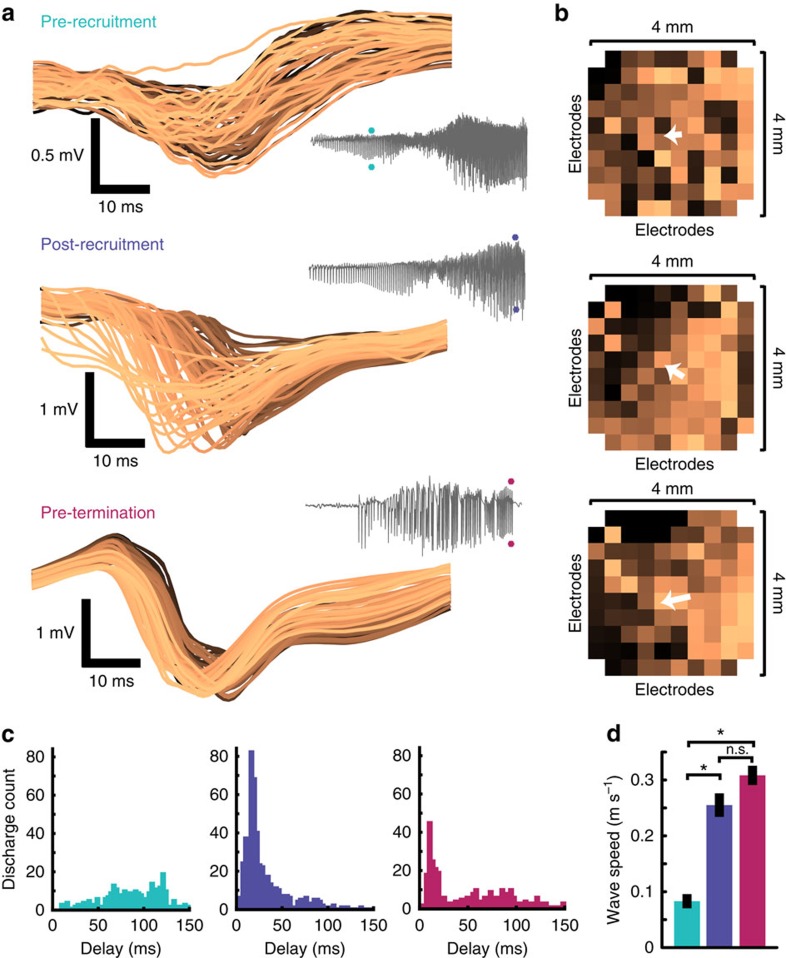
Ictal discharges form travelling waves across the microelectrode array. (**a**) Example, low-frequency LFP recorded from each microelectrode during three ictal (EEG) discharges, one from each seizure epoch as indicated by the coloured asterisks in the seizure traces, colour coded by when the maximally negative slope of the travelling wave occurs on each microelectrode. Seizures from two patients (patient 3 and 5) are shown. (**b**) The footprint of the microelectrode array corresponding to the three ictal discharges in **a**, with electrode positions colour coded the same way as the LFP in **a**. Vectors indicating travelling wave direction are superimposed on the microelectrode array footprint in white. (**c**) Histograms of delays between the first and last discharges on array microelectrodes during each epoch (pre-recruitment in light blue, post-recruitment in purple and pre-termination in pink). (**d**) Measures of discharge speed across all patients, during the three epochs shown in **a**. *Post hoc* Tukey's range test determined that pre-recruitment bursts (*N*=273) were significantly slower than both post-recruitment (*N*=315) and pre-termination (*N*=282) bursts (*P*=9.5 × 10^−10^ and *P*=9.5 × 10^−10^). Post-recruitment and pre-termination bursts did not exhibit significantly different speeds (*P*=0.82). Error bars indicate s.e.

We examined ictal discharges individually in the MEA recordings, to assess their spatiotemporal evolution on the fine (400 μm) scale afforded by the MEA configuration. Analysis of low-frequency (<50 Hz) LFP during ictal discharges consistently revealed variable latencies across the MEA (Fig. 2a). The direction of travel of each discharge was determined by ranking the minima of the first derivative (that is, the maximally decreasing slope) of the low-frequency LFP in time. Using these ranked minima, a velocity vector was defined for each discharge, indicating the direction taken by the travelling wave (Fig. 2b). The extent of the measured delays across the MEA, which along its maximum diagonal spans 5.67 mm, was 25.96±17.03 ms (mean±s.d.; range=4–135 ms; Fig. 2c). Travelling wave speeds estimated from these delays were 0.21±0.31 m s^−1^ (mean±s.d.; range=0.42–1.42 m s^−1^). Corresponding latencies were noted in the associated MUA bursts (Supplementary Fig. 6a,b). These latencies determined from MUA were not significantly different from those determined from low-frequency LFP (Supplementary Fig. 6c,d; repeated measures analysis of variance, *P*=1).

Examining travelling wave speeds in each epoch revealed that speeds increased significantly from the pre-recruitment to post-recruitment and pre-termination epochs (mean±s.d. speed for pre-recruitment: 0.086±0.07 m s^−1^, post-recruitment: 0.26±0.24 m s^−1^, pre-termination: 0.31±0.17 m s^−1^; analysis of variance with Tukey *post hoc* tests, *P*<0.01; Fig. 2c), as measured by both the magnitude of the velocity vector (Tukey *post hoc* test, *P*<0.01; Supplementary Fig. 3) and travelling wave delays across the array (Tukey *post hoc* test, *P*<0.01; Supplementary Fig. 4). This speed change is consistent with the collapse of inhibition at the ictal wavefront. After recruitment, travelling wave speed remained fairly constant, with no significant difference between the post-recruitment and pre-termination epochs (Tukey *post hoc* test, *P*=0.31).

### Travelling waves arise from the ictal wavefront

Previous studies from mouse neocortical slices *in vitro* showed that ictal discharges propagate away from the ictal wavefront along the cortical space in a brain slice^15^. The hypothesis follows that an analogous propagation pattern of fast-moving synaptic potentials arises from the ictal wavefront, as it slowly and radially expands across the two-dimensional cortical sheet (see Supplementary Movie 2 in Schevon *et al*.^13^ for a clear example of ictal wavefront propagation across the MEA). To test this hypothesis, we first determined the direction of propagation of the ictal wavefront from the propagation of asynchronous multiunit firing across the MEA. We then examined the directions of rapid travelling waves, relative to the direction of the slow ictal wavefront, for each discharge during the pre- and post-recruitment epochs and the pre-termination epoch.

**Figure 3:**
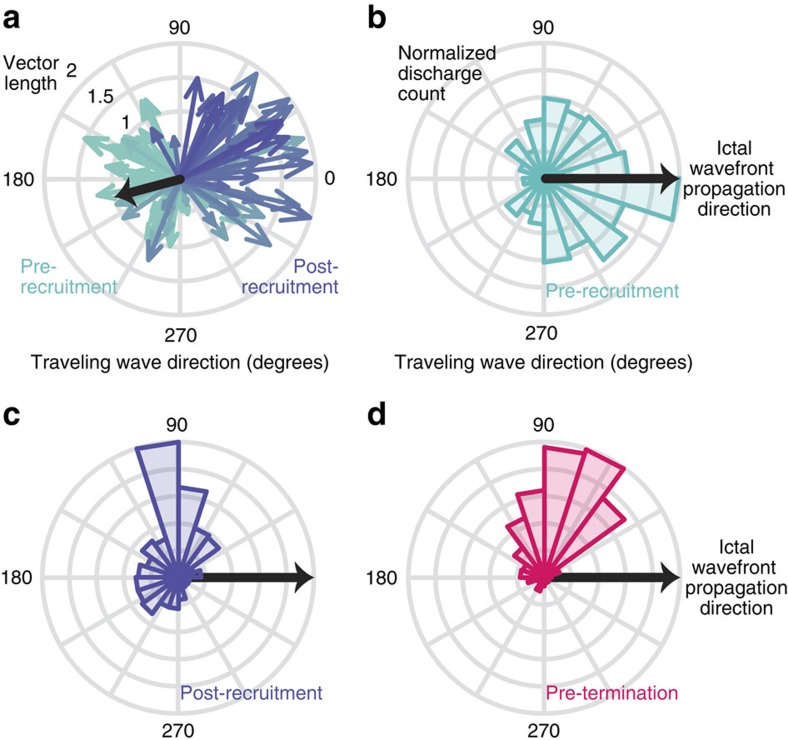
Travelling wave directions during seizure epochs are dependent on the location of the ictal wavefront. (**a**) Example velocity vectors for one seizure before and after recruitment. The black line indicates the direction of ictal wavefront passage across the MEA. The shift in direction between pre-recruitment and post-recruitment discharges that occurs precisely at the time of ictal wavefront passage is apparent. In each case, the ictal wavefront's location relative to the MEA site is opposite to the predominant travelling wave direction. (**b**–**d**) Distributions of travelling wave directions across all seizures, relative to the direction of the ictal wavefront (black line), coloured by epoch (**b**: pre-recruitment, **c**: post-recruitment, **d**: pre-termination).

Travelling waves in each epoch showed statistically significantly preferred directions across the spatially fixed layouts of each MEA and ECoG grid (Rayleigh's test of non-uniformity, all *P*<0.01). Two predominant, opposing directions relative to the propagation direction of the ictal wavefront were observed across the MEA (Fig. 3a). Travelling wave directions before and after passage of the ictal wavefront through the MEA were significantly different both within (Kuiper's test, *P*<0.01) and across (Kuiper's test, *P*<0.01) patients. Pre-recruitment travelling waves were aligned with the direction of the slow-moving ictal wavefront (one-sample test for mean angle, all *P*>0.05; Fig. 3b). Post-recruitment and pre-termination travelling wave directions were oriented in the opposite direction of the slow movement of the ictal wavefront (one-sample test for mean angle, *P*<0.01 for travelling waves in both post-recruitment and pre-termination epochs; Fig. 3c,d).

This perceived flip in travelling wave direction from the vantage point of the fixed cortical recording site of the MEA, coinciding with ictal wavefront passage, indicates that synaptic travelling waves during ictal discharges originate from the ictal wavefront in space and time. Specifically, before the arrival of the ictal wavefront at the MEA, discharges travelled in the same direction as the ictal wavefront. After ictal wavefront passage, the discharges abruptly changed direction, that is, were directed back into the ictal core (Supplementary Movie 2).

**Figure 4:**
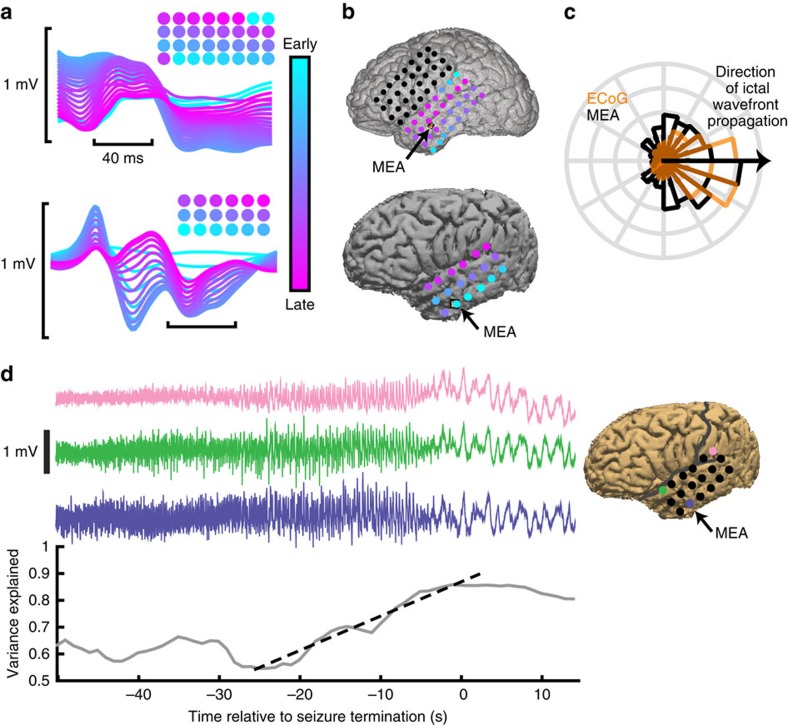
Travelling waves propagating across the microelectrode array correspond to those propagating across the ECoG grid. (**a**) Examples of ictal discharges recorded across ECoG electrodes in two patients colour coded by when the maximally decreasing slope occurs, with cyan indicating earlier electrodes and magenta later electrodes. Colour-coded circles above the discharges indicate a spatial representation of delays over the ECoG grid, with the same colour code as the voltage traces. (**b**) Reconstructions of ECoG grid and MEA locations for the same patients in **a**. Average delays across ECoG electrodes are colour coded as in **a**. Black arrows indicate MEA locations. (**c**) Distributions of MEA pre-recruitment travelling waves (black) and travelling waves ECoG (orange). (**d**) Three channels of LFP recorded from ECoG electrodes during one seizure (patient 3) and a representative increase in variance explained by the first-principal component, across all ECoG electrodes on the grid shown in **b** (lower), during the pre-termination epoch. The black dotted line shows linear regression used to determine the slope of the variance explained during the pre-termination epoch for the example seizure.

Travelling waves during ictal discharges were also observed extending across the ECoG (subdural) grid. Figure 4a shows examples of ictal discharges recorded across ECoG electrodes in two patients, colour coded by when the maximally decreasing slope occurs, and the ranked delays across ECoG electrodes. These travelling waves across ECoG electrodes also showed consistent directions within each seizure (Rayleigh's test of non-uniformity, all *P*<0.01), and matched the direction of travelling waves seen in the simultaneous MEA recording. Figure 4b shows the median ranks of travelling wave delays superimposed on brain reconstructions for patients 3 and 5. Travelling waves on ECoG appeared to extend from the ictal wavefront as well, having overlapping relative direction distributions with pre-recruitment waves on the MEAs (Fig. 4c; Kuipers test, all *P*≥0.1). Low-frequency LFP was also increasingly synchronized across the ECoG grid as seizures approached termination (mean±s.d. slope of linear regression: 0.011±0.0056; Fig. 4d). This pre-termination synchrony increase was apparent in 15 out of 17 seizures examined (Supplementary Fig. 4). These results show that rapidly propagating travelling waves from the ictal wavefront are also apparent across ECoG electrodes, where they give rise to the commonly observed ‘hypersynchrony' during seizures.

### Simulated ictal wavefront reproduces termination seizure dynamics

That the speed and direction of travelling waves during ictal discharges are dependent on the relative location of the ictal wavefront suggests the ictal wavefront is the agent of both seizure propagation and termination. The hypothesis follows that a dissipation, weakening or cessation of the ictal wavefront should result in seizure termination. To acquire a theoretical understanding of how input from the ictal wavefront affects the neural dynamics of the ictal core, we examined a simple mean-field computational model of the ictal core. Specifically, we modelled the contribution of the ictal wavefront as input to a small recurrently connected network of simulated neurons. The model used experimentally determined parameters of layer 5 pyramidal neurons. The specifics of the model, including these parameters, are described in detail in Supplementary Information. Similar approaches have been used as simple models of spinal cord neuron dynamics and working memory traces^31^,^32^.

**Figure 5:**
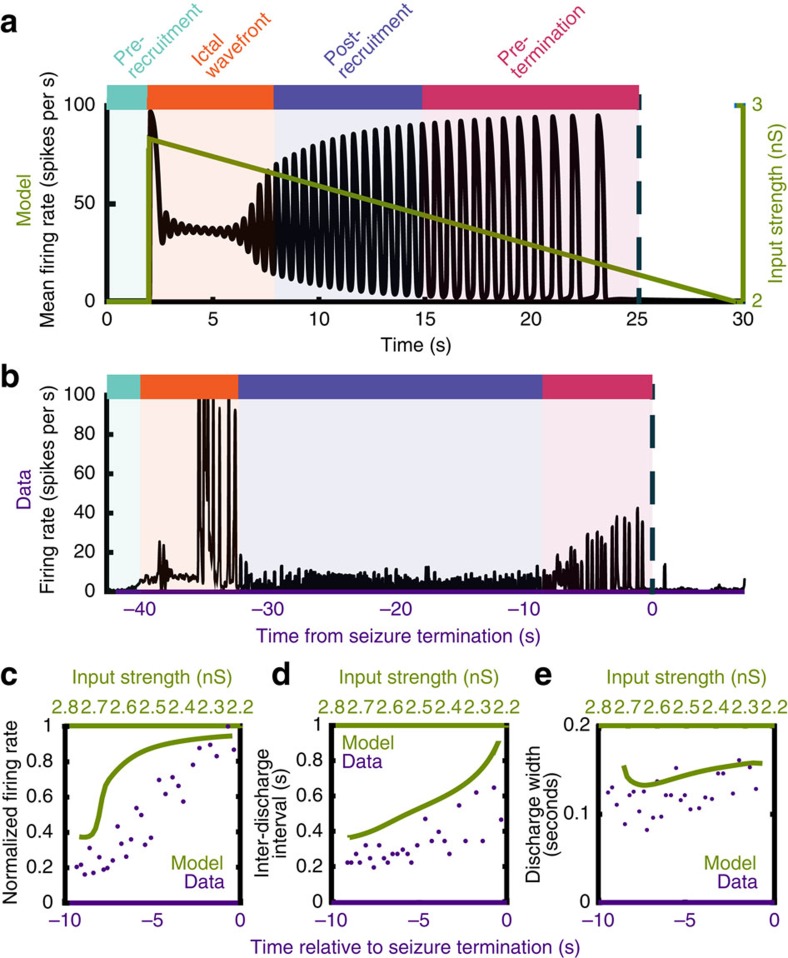
Decreasing input to a network of simulated neurons recreates mean-field dynamics observed in the data. (**a**) Mean firing rate of the simulated ictal network progresses from tonic firing, to rhythmic discharging, to self-termination, with decreasing extra-network input. The colours correspond to the epochs labelled in Fig. 1 as apparent in the model's firing rate. (**b**) Firing rate from all microelectrode channels during one spontaneous human seizure, showing the progression from tonic firing during ictal wavefront passage, to rhythmic discharging, to seizure termination. The coloured bars above the seizures correspond to the epochs labelled in Fig. 1. (**c**) Peak mean firing rate during each discharge in the pre-termination period for the simulated neurons (green) and the seizure shown in **b** (purple). (**d**) Inter-discharge intervals during the pre-termination period, for the simulated neurons (green) and the seizure shown in **b** (purple). (**e**) Discharge width during the pre-termination period, for the simulated neurons (green) and the seizure shown in **b** (purple).

The qualities of the MUA dynamics we observed in the ictal core (Fig. 5a,b) were reproduced well in our model by simply decreasing the external input to the simulated network, as would happen with a gradual dissipation of the ictal wavefront's excitatory drive. Specifically, with high external input, a seizure-like pattern was initiated and the model was locked into a tonically firing state. As input decreased, the model activity first underwent a transition from tonic firing to a pattern of rhythmic discharging. As input continued to decrease, peak firing rate, IDI and discharge width all increased steadily until the seizure terminated with an abrupt cessation of discharging. These trends were present both in the model and in the recorded seizure data (Fig. 5c–e).

### Signatures of a weakening ictal wavefront

**Figure 6:**
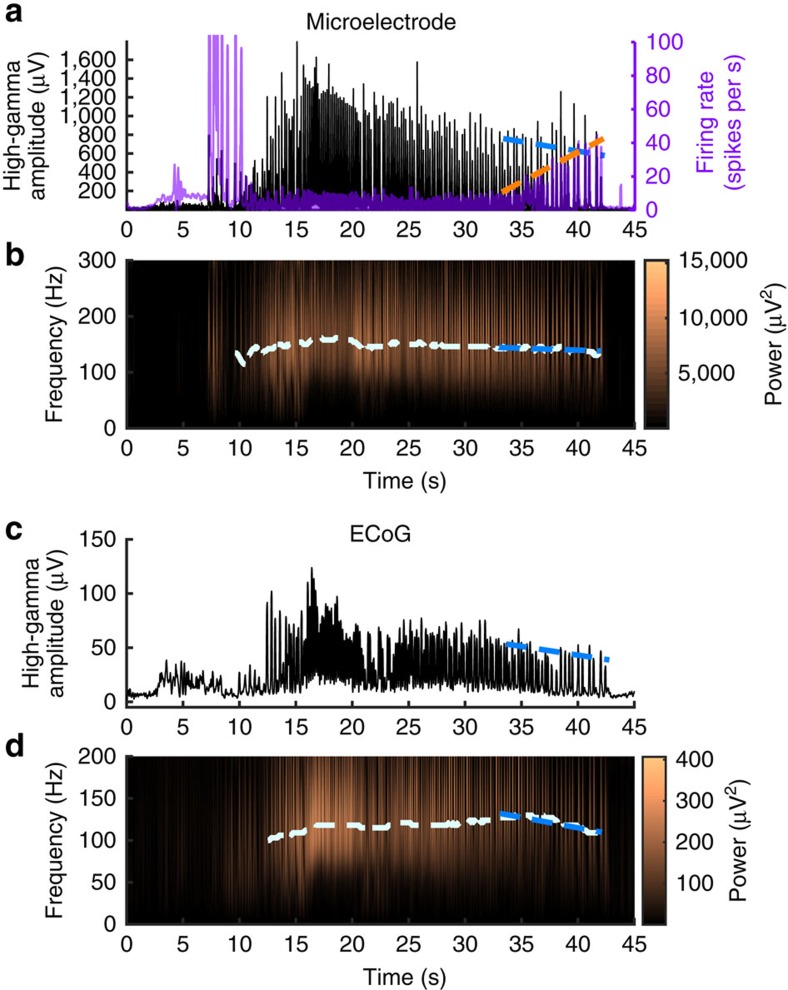
High-γ activity in the seizure core decreases in amplitude and frequency before seizure termination. (**a**) High-γ instantaneous amplitude and multiunit firing recorded from an example microelectrode through the duration of the same seizure shown in Fig. 5b. The blue dotted line indicates linear regression of the peak high-γ amplitude for each discharge during the pre-termination epoch, and the orange dotted line similarly indicates peak firing rate. (**b**) Spectrogram of the same seizure in **a**. The white dotted line indicates peak high-γ frequency through the duration of the seizure, and the blue dotted line indicates linear regression of the peak high-γ frequency during the pre-termination epoch. **c** and **d** show the same seizure as in **a** and **b** recorded on the ECoG electrode adjacent to the microelectrode array. Dotted lines indicate the same linear regression measurements as in **a** and **b**.

We next sought to quantify the predicted effects of our hypothesized seizure spread and termination model in multi-scale electrophysiological recordings of human seizures. Due to the effect of seizure recruitment on action potential waveforms^33^, multiunit events were detected by thresholding the filtered (500–3000 Hz) MEA data as in prior publications^13^,^14^,^33^. To assess whether the predicted increase in multiunit firing rates towards seizure termination were present in the ictal core, we quantified trends in peak MUA and high-γ amplitude during the pre-termination epoch using linear regression. Linear regression slopes were tested against a zero-slope null hypothesis to determine significance of trends in activity leading up to seizure termination. In the ictal core, peak MUA averaged across all recorded microelectrode channels increased significantly as the seizure progressed from recruitment to termination (Wilcoxon signed-rank test, *P*<<0.01; that is, Fig. 6a). In contrast, no significant trend in peak MUA was observed across MEA channels in the penumbra before seizure termination (Wilcoxon signed-rank test, *P*=0.41; Supplementary Fig. 1c).

Our hypothesis predicts that synchronization of unit firing gradually decreases in the ictal core as a seizure approaches termination. This trend occurs despite the increase in multiunit firing rate. In the MEA data, there was a significant decrease in the high-γ envelope across MEA channels in the ictal core (Wilcoxon signed-rank test, *P*<<0.01; Fig. 6a). In contrast, high-γ recorded at penumbral MEA sites exhibited a significant increase in pre-termination high-γ power (Wilcoxon signed-rank test, *P*<<0.01; Supplementary Fig. 1b). Peak high-γ frequency also decreased in seizures recorded from the ictal core from 183.9±25.4 Hz at the beginning of the post-recruitment period to 141.5±16.1 Hz at seizure termination (Wilcoxon signed-rank test, *P*<0.01; Fig. 6b).

Although the MEA data are highly specific, they can provide information only from the 4 × 4-mm coverage area. The clinical ECoG grid, on the other hand, samples a region spanning several centimetres, but with lower (one cm) spatial resolution. While MUA is not accessible from these large subdural electrodes, the location of the ictal core can be inferred from high-γ activity recorded on ECoG, which acts as a surrogate for intense neuronal bursting^14^. Paralleling the MEA recording, we expected to find decreases in high-γ frequency and amplitude across clinical ECoG electrodes that exhibited high-γ activity indicative of recruitment into the ictal core. We therefore tested all ECoG recording sites at which the high-γ amplitude exceeded five times the s.d. of pre-ictal recordings (15.9±15.0 electrodes per seizure). High-γ amplitude decreases were observed in these ECoG electrodes in all five patients, including the two patients with the MEA positioned outside the ictal core (Wilcoxon signed-rank test, *P*<0.01; Fig. 6c), with decreases noted individually in 15 of 17 seizures. High-γ peak frequency also decreased in recordings from the same ECoG electrodes during the pre-termination period, from 138.5±29.9 to 109.2±32.1 Hz (Fig. 6d).

### Pre-termination multiunit and high-γ activity correlation

**Figure 7:**
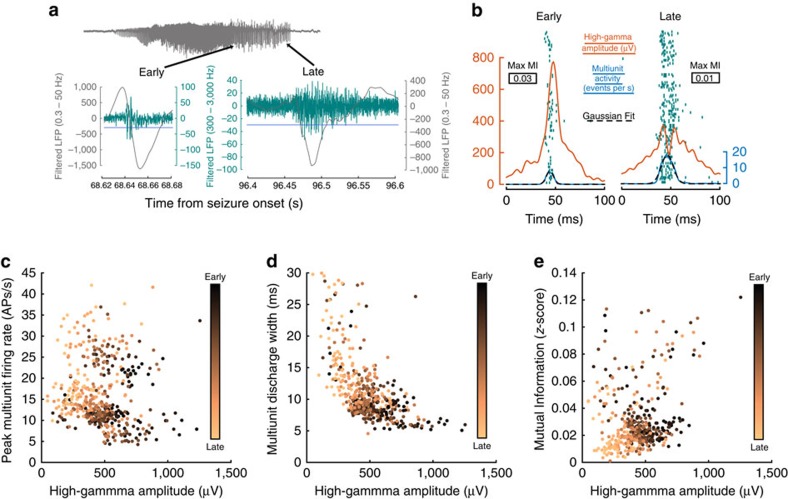
Local desynchronization in the ictal core precedes seizure termination. (**a**) Examples of filtered data for two sample discharges during the pre-termination epoch. Low-frequency filtered data (0.3–50 Hz) are shown in grey and filtered data for multiunit activity detection (500–3000 Hz) are shown in teal. The threshold for multiunit event detection is indicated with a blue line. Bursts are indicated on the seizure in grey using black arrows. (**b**) Examples of high-γ (orange), firing rate (blue) and Gaussian fit (black dotted lines) for burst width superimposed on the multiunit activity raster plot (teal) for two bursts in one seizure: one at the beginning of the pre-termination epoch (early) and one at the end of the pre-termination epoch (late). Maximum mutual information values for each discharge are shown in boxes. (**c**) Relationship between peak multiunit firing rate and peak high-γ amplitude for each discharge. (**d**) Relationship between high-γ amplitude and multiunit discharge width for each discharge. (**e**) Relationship between peak high-γ amplitude and multiunit synchrony in each discharge. Colours in **c**, **d**, and **e** represent when each discharge occurred during the pre-termination epoch, with black dots occurring earlier and copper dots occurring later.

The progressive dissociation in the trends between high-γ and firing rate, as a seizure approaches termination, indicates that the previously observed correlation between the two signals^34^,^35^ may have multiple contributing factors. It has been proposed that action potential synchrony provides a substantial contribution to the amplitude of the high-γ signal^36^,^37^,^38^. To address the relative contribution of action potential synchrony to high-γ amplitude, correlations among peak multiunit firing rate, peak high-γ amplitude and multiunit discharge width were examined among individual discharges during the pre-termination epoch. Examining these metrics for each discharge controls for the variable intervals between successive discharges and allows for measurement of synchrony over discrete time periods, defined by the natural progression of the seizure. Again, there was a significant anti-correlation between per-discharge firing rate and high-γ amplitude as would be expected from the aforementioned trend (Spearman's *ρ*=−0.21, *P*<0.01; Fig. 7b). However, a greater anti-correlation was observed between per-discharge high-γ amplitude and the multiunit discharge width (Spearman's *ρ*=−0.60, *P*<<0.01; Fig. 7c), which was defined as the s.d. of a Gaussian fit to the MUA during each discharge (Fig. 7a). This result suggests that the degree of temporal dispersion of MUA is correlated with the amplitude of the high-γ signal recorded across the MEA.

To examine the relationship between multiunit synchrony and high-γ amplitude explicitly, mutual information for multiunit event coincidence was measured among all pairs of microelectrodes during the pre-termination epoch. Mutual information was significantly correlated with high-γ amplitude over discharges (Spearman's *ρ*=0.38, *P*<<0.01; Fig. 7d). Together, these results show that action potential firing in the ictal core desynchronizes as a seizure progresses towards termination. Desynchronization therefore manifests as decreasing high-γ amplitude and peak frequency in both MEA and ECoG recordings.

## Discussion

**Figure 8:**
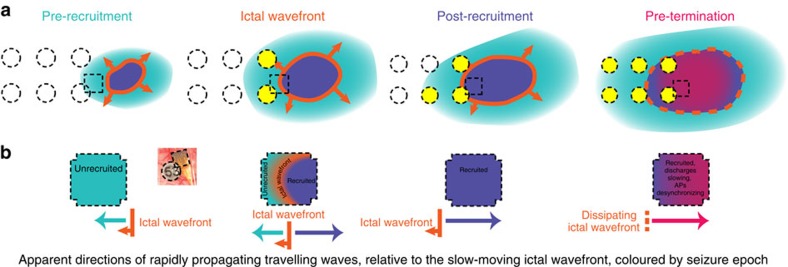
Summary model of spatial dynamics of seizure evolution and termination. (**a**) Schematic showing snapshots of seizure progression through the four seizure epochs. Colours represent the spatial organization of the epochs shown in time series data of Fig. 1. Dashed-line squares represent the MEA footprint, and dashed-line circles represent ECoG electrodes. Yellow ECoG electrodes represent those exhibiting hypersynchronous ictal activity in the low-frequency (0.3–50 Hz) LFP. As the recruited region (seizure core) gradually expands to envelope the MEA site, the ictal wavefront approaches the site, passes through it and then continues to move away from the site. (**b**) Schematic depictions of travelling wave activity across the MEA microelectrodes during each of the four seizure epochs. Rapidly moving travelling waves across the MEA change direction based on the MEAs location relative to the slowly moving ictal wavefront.

The analyses of spontaneous human seizures and simulations of ictal neurons presented here support the hypothesis that the migrating ictal wavefront is the primary source of ictal activity, and consequently also the agent of seizure termination (Fig. 8). Thus, the seizure is sustained by activity in newly recruited territories, much like a forest fire is sustained by fresh wood. We describe a straightforward electrophysiological mechanism that is sufficient to explain the major electrophysiological hallmarks of populations of neurons in the ictal core leading up to seizure termination: increased multiunit firing rate, desynchronization of MUA, decreases in both high-γ amplitude and frequency, changes in the directions of travelling waves of ictal synaptic activity, and a slowing rate and increasing width of ictal discharges. An important conclusion is that the wide-ranging effects of seizures appear to be triggered from a small cortical region, which migrates during the seizure. That is, the sustenance and eventual termination of the seizure may depend on the intense activity of small, spatially restricted populations of neurons. The simultaneous, wide-area seizure termination can therefore be parsimoniously explained by sufficient dissipation of input from the ictal wavefront.

Previous studies have shown increasing low-frequency synchronization towards the end of seizures^3^,^5^ and point to hypersynchrony as a primary electrophysiological hallmark of seizures^7^,^39^. On the basis of these observations, it has been proposed that seizures are mediated by a large-scale network structure with coordinated long-range neuronal interactions^2^,^5^,^40^. This model would suggest that the MUA associated with ictal discharges should also demonstrate increasingly tight synchronization over extended cortical territories. We found that the opposite is the case. Further, the large-scale field potential synchronization in ECoG recordings can be explained as a by-product of travelling waves moving rapidly across large areas of cortex (Fig. 4). We theorize that these travelling waves resemble those previously studied under normal conditions: both field potential and multiunit firing components are generated locally in response to transmitted synaptic barrages^25^,^27^. The rapid travelling wave speeds, up to 1.42 m s^−1^, together with the relatively long (up to 500 ms) duration of ictal discharges^29^,^41^, result in substantial temporal overlap and thus high levels of measured synchronization. Furthermore, as IDIs increase leading up to seizure termination, there are gaps between discharges that are markedly more synchronous than the discharges themselves (Supplementary Fig. 5).

The discrepancy between the apparent increases in LFP synchrony and the decreases in MUA synchrony proved to be a consistent feature of seizures regardless of recording modality or scale. While multiunit firing bursts were aligned with ictal discharges, these bursts became gradually desynchronized as the seizure approached termination—despite a corresponding increase in firing rate. Although multiunit desynchronization could only be directly observed in the area sampled by the MEA, indirect evidence of the same process was noted across a much wider area: correlated with the decreasing multiunit synchrony was a decline in high-γ amplitude and frequency that was present both in the microelectrode recordings and in the ECoG recorded from nearby clinical subdural electrodes. Accordingly, we observed that all clinical ECoG recordings in our study sample demonstrating significant ictal high-γ activity showed similar decreases in high-γ amplitude and frequency, indicating that the desynchronization process observed in the MEA recordings occurs throughout the recruited cortex.

The local neuronal population dynamics of the ictal core, including the increasing IDIs leading up to seizure self-termination, can be explained as the effect of gradually decreasing excitatory input from the ictal wavefront. The computational model presented here indicates that a dissipating or weakening ictal wavefront is sufficient to produce all the observed temporal dynamics of the ictal core, including self-termination, across a wide cortical region. The ictal wavefront is <2 mm wide^13^,^23^ because its intense tonic firing can only be sustained by very-high-intensity excitatory input that results from the failure of strong feedforward inhibition. Furthermore, the model predicts that only a slight drop in excitatory drive is sufficient to end the tonic firing phase and for the local neuronal population to convert to a repetitive bursting pattern^42^,^43^. The model predicts that further decreases in input actually increase firing rate up until the point where bursting stops abruptly. All of these dynamics are present in MUA recorded from the ictal core in spontaneous human seizures.

Future experimental and modelling work will be needed to characterize the ictal wavefront's reduced input mechanism fully, as these passive recordings in epilepsy patients undergoing clinically necessitated monitoring do not permit destructive experimental manipulations. For example, while the current study provides evidence that paroxysmal discharges originate from the ictal wavefront, there is little data apart from computational studies^42^,^43^ addressing how neurons undergo a transition from intense tonic firing to patterns of rhythmic discharging. Although the current results support our hypothesis that input from the ictal wavefront weakens in the pre-termination epoch, the mechanisms of this weakening remain to be determined. For instance, the wavefront may run into a ‘blind alley', surrounded by tissue that is already recruited and becoming increasingly refractory, or it may encroach onto other areas that have a superior inhibitory restraint. The detailed characterization of pre-termination seizure electrophysiology we have provided, however, can be used to direct experimental manipulations capable of testing these hypotheses.

Many proposed mechanisms for seizure termination in acute animal studies operate over a limited cortical region^44^, leaving open the question of how they could subserve large-scale, simultaneous seizure termination. These mechanisms include acidosis^11^, changes in intracellular and extracellular ion concentrations^7^,^45^, increased membrane potassium conductance^46^,^47^, suppression from subcortical structures^48^,^49^, and neurotransmitter^50^ or ATP depletion^51^,^52^. The thesis presented herein implies that much of observed ictal electrophysiological activity, both core and penumbral, occurs as a response to electrical signals broadcast from the ictal wavefront. While these results do not directly implicate a specific biophysical mechanism for human focal seizure termination, they provide fresh insights into the processes leading up to seizure termination in humans and explain how cellular-scale processes can be compatible with clinical EEG observations.

A salient finding presented here is the progressive dissociation between high-γ and MUA leading up to seizure termination. High-γ activity, that is, high-frequency, broadband LFP ranging from ∼70 to 300 Hz, depending on the electrodes and amplifiers used to record the electrical signals^53^,^54^, is functionally distinct from band-limited gamma (30–50 Hz) oscillations and is thought to reflect summated postsynaptic output from the local neuronal population^36^,^55^,^56^ in a submillimetre domain^20^,^57^. High-γ power has been shown to be highly correlated with firing rate in studies of evoked visual stimuli in monkey visual cortex^38^, and in microwire recordings from the human medial temporal lobe^35^ or neocortically implanted microelectrodes and standard clinical electrodes^14^,^58^,^59^,^60^. In contrast, our results indicate that changes in action potential synchrony, independent of firing rate, can affect the high-γ signal. However, further investigation of this effect in normal burst firing versus epileptic bursting is warranted.

It has been proposed that population action potential synchrony is an important contributing factor to high-γ power^36^,^37^. Furthermore, simulations show that increased action potential synchrony increases LFP power, especially in the high-γ range^42^,^61^. The current results support these mechanistic understandings of high-γ by showing, in spontaneous human recordings, a decrease in multiunit synchrony that is highly correlated with a decrease in high-γ amplitude and peak frequency—despite marked firing rate increases towards the end of a seizure. Both the increased firing rate and the decreased multiunit synchronization may be a consequence of the increased IDIs in the pre-termination phase. As discharges in a seizure separate temporally, there is a larger window for action potential firing to occur before the next discharge, or more time for fast metabolic and ionic recovery. In this scenario, more action potentials could fire with greater temporal dispersion because there is a larger time window in which to do so. The strong relationship between MUA synchrony and the high-γ amplitude suggests that action potential synchrony provides a substantial contribution to the high-γ signal.

Finally, our results have implications for treatment and prevention of epilepsy. Therapies targeting the source of seizures have always been hampered by the lack of knowledge of what this is precisely. Our results suggest novel avenues for improving the detection and localization of sites that drive seizure activity using the speed and direction of travelling waves to localize the ictal wavefront. Recent developments in high-density recording technology have provided the means to record many single units across the surface of the human brain from a thin-film substrate^62^. Such recordings could detect travelling wave directions, as well as the ictal wavefront and core, over broad cortical areas with high spatial resolution.

Future investigations into the mechanisms of ictal wavefront dissipation and seizure termination could yield new approaches for therapies to terminate seizures earlier, by hastening the dissipation of the ictal wavefront, for example. Stopping seizures earlier could greatly reduce the burden of epilepsy, as longer seizures have been associated with more severe postictal paralysis^63^, peri-ictal respiratory dysfunction^64^ and are likely associated with more severe postictal maladies in general.

The current results support an analogous model of human seizure propagation to that observed in brain slice experiments and examine the model's implications for the mechanisms of seizure termination. We have shown that local desynchronization leading up to seizure termination is accompanied by a breakdown in the commonly observed correlation between action potential firing and high-γ activity. Local desynchronization in the ictal core occurs despite increasing large-scale synchronization of low-frequency LFP across the brain, which is a by-product of rapidly travelling waves originating at the ictal wavefront. These results describe the spatiotemporal structure of seizure generation and termination in human chronic epilepsy and have implications for targeted treatments aimed at stopping seizures.

## Methods

### Patients and ethics statement

Study participants consisted of adults with pharmacoresistant focal epilepsy who underwent chronic invasive EEG studies to help identify the epileptogenic zone for subsequent removal. During this process, these patients had arrays of ECoG electrodes implanted across large sections of their brains to localize their seizure focus. These patients also had a 4 × 4 mm MEA with 96 (10 × 10 electrode grid) 1-mm long penetrating microelectrodes (MEA, Blackrock Microsystems, Inc., Salt Lake City, UT) implanted into the clinically defined seizure onset zone, along with the subdural clinical ECoG electrodes. Histology work has previously confirmed that these electrodes were positioned in layers 4 and 5 in the human-association cortex^13^. However, it is likely that there is some variability due to individual variations in cortical topology, as well as to tissue changes stemming from the implant itself. House *et al*.^65^ provide a detailed description of the surgical procedures for implanting this particular type of MEA into humans. The study was approved by the Institutional Review Boards of the Columbia University Medical Center and University of Utah, and informed consent was obtained by each patient before implantation.

Signals from the MEA were acquired continuously at 30 kHz per channel (0.3 Hz–7.5 kHz bandpass, 16-bit precision, range ±8 mV). The reference was either subdural or epidural, chosen dynamically based on recording quality. Subdural EEG signals were acquired using a standard clinical video EEG system (XLTek, Natus Medical Inc., Oakville, ON, Canada) at 500–2000 Hz per channel (0.5 Hz high pass, low-pass set to ¼ sampling rate, 24-bit precision), and then subsequently downsampled to 500 Hz. The two data sets were aligned using a pulse-coded signal delivered simultaneously to digital input ports of both recording systems.

Although all of the MEAs examined in this study were implanted into the clinically defined seizure onset zone, none recorded seizure initiation, and only three MEAs were recruited into the locally seizing territory, termed the ictal core. The ictal core, defined as the locus of intensely discharging neuronal firing during seizures, was identified as described in ref. 13. Six seizures from three patients in which the MEA was positioned within the ictal core were examined. In one of these seizures, the MEA recording (but not the ECoG recording) was interrupted after recruitment into the seizure and before seizure termination. This seizure is therefore excluded from direct analyses of the pre-termination epoch. Ten seizures from two patients in which the MEA was not recruited into the ictal core were also examined.

### Data collection and preprocessing

As sorting single units is not possible in the ictal core due to the loss of recognizable waveforms after ictal wavefront passage^33^, timestamps for MUA were instead derived using previously established detection methods^13^. MUA was segregated from the raw LFP by band-pass filtering the raw LFP between 500 and 3,000 Hz using a 150th order finite impulse response (FIR) filter. Negative peaks in this signal were detected and those peaks that exceeded four times the s.d. of the signal in the negative direction, and that occurred >1 ms after immediately preceding peaks, were retained as multiunit timestamps and waveforms. Any peaks that exceeded eight times the s.d. of the filtered MUA signal were rejected as artefact. Firing rate was calculated from these multiunit timestamps in 100-ms windows every 25 ms through the duration of the seizure.

High-γ was segregated from the raw LFP by band-pass filtering the raw LFP between 50 and 300 Hz for MEA data and 50 and 150 Hz for ECoG data, based on acquisition sampling rates, using a 150th order FIR filter. The lower range of this interval was chosen to include the low high-γ frequencies observed towards the end of seizures. High-γ instantaneous amplitude was defined as the absolute value of the Hilbert transform of this signal. To examine LFP spectral content, instantaneous amplitude and phase were first extracted by Morlet wavelet decomposition on 84 scales from 1 to 300 Hz for MEA data and 1 to 150 Hz for ECoG data. The absolute values of these wavelet decompositions were then normalized by the inverse of the frequency spectrum analysed.

### Natural epochs in human seizures

To understand the dynamics of the ictal core leading up to seizure termination, it was necessary to identify transitions local ictal activity. Accordingly, the MEA seizure recordings were segmented into four distinct epochs between the fixed times of seizure onset and termination. Seizure onset and termination were determined clinically from the low-frequency ECoG, by consensus of two epileptologists (C.A.S. and L.M.B.).

The ictal wavefront epoch was determined by smoothing the firing rate of each channel with a Gaussian kernel with a 200-ms s.d. The width of this kernel emphasized the tonic firing of the ictal wavefront and de-emphasized the faster bursting activity of the ictal core. The ictal wavefront epoch was defined as the time of the first channel's mean minus its s.d. until the last channel's mean plus its s.d. The pre-recruitment period was operationally defined as the time of seizure onset until the start of the ictal wavefront epoch. The post-recruitment and pre-termination periods comprised the interval between the end of the ictal wavefront epoch and seizure termination. The division point between the post-recruitment and pre-termination epochs was determined by calculating the coefficient of variation over the IDIs after end of the ictal wavefront epoch. The coefficient of variation was calculated as the s.d. of groups of 30 IDIs divided by the mean of those IDIs, sliding 3 IDIs at a time through the duration of the seizure. If the coefficient of variation exceeded 5% for any group of 30 IDIs, then the time of the first IDI in that group defined the division between post-recruitment and pre-termination epochs.

### Travelling waves and large-scale LFP synchrony

The direction of the travelling wave in each discharge was determined by ranking MEA or ECoG channels according to the time of occurrence of each ictal discharge, using the broadband LFP signal. Time of occurrence was measured using the minimum of the first derivative of the low-frequency LFP (<50 Hz); that is, the point of maximally decreasing slope. A similar method, which emphasizes fast components of the discharge, has been previously employed to detect interictal discharge propagation^15^,^28^. Positive and negative weighted centroids were then determined for the resulting channel rankings across the MEA or ECoG grid footprint space. The resultant of these two points defined the velocity vector for the travelling wave. Rayleigh's test for non-uniformity was used to determine whether the vectors were normally distributed on the unit circle. Kuiper's test was used to compare distributions of wave direction angles. Descriptive circular statistics were calculated using the circular statistics toolbox for Matlab^66^.

The direction of the ictal wavefront and directions of travelling waves were determined in similar ways, but with different temporal granularity. The peak firing rate (calculated using the 200 ms kernel mentioned above) was ranked for each channel during the ictal wavefront epoch and the centroid method mentioned above was carried out on these peaks to calculate propagation direction for the ictal wavefront. Directions of individual travelling waves could also be determined from MUA in a similar manner, but using a kernel with a 10-ms s.d. to emphasize the fast ictal discharges (Supplementary Fig. 6).

Broadband LFP synchrony was evaluated in a similar manner to previous studies^67^. Principal component analysis was performed on 1 s of data, from all ECoG electrodes on the subdural grids overlying the MEAs, every 10 ms through the duration of the seizure. Synchrony was measured as the amount of variance explained by the first-principal component (that is, how much variance in the ECoG signal can be explained by a single covariance dimension). Trends in the slopes and mean values of this measure of synchrony were quantified during the pre-termination epoch using linear regression.

### Ictal core and wavefront simulation

To gain a theoretical understanding of the ictal wavefront on seizure electrophysiology, the relatively small network of reactive excitatory cells in the ictal core was simulated using a mean-field approach based on previous computational studies^31^,^32^. The simulated ictal core had recurrent, intra-network and extra-network inputs. Intra-network input was subject to firing rate-dependent synaptic depression, as in previous studies^31^,^32^,^61^. The extra-network input was used to model the contribution of the ictal wavefront to the network of cells in the ictal core. Extra-network input was linearly decreased in a biophysically plausible range for seizure activity, and the temporal dynamics of the simulated ictal core were observed. Details of the model, including cellular parameters (Supplementary Table 1), can be found in Supplementary Information.

### Per-discharge analyses

Due to the typical ictal pattern featuring trains of transient high-amplitude discharges with relative attenuation of signal in between, all analyses were conducted on a per-discharge basis. Ictal discharges were first isolated by identifying all local maxima in both mean high-γ amplitude and mean multiunit firing rate across MEA channels that exceeded twice the s.d. above the mean computed from the entire seizure. Some peaks in multiunit or high-γ activity appeared during the seizure without a peak in the other signal. The operational definition of discharges in the ictal core therefore required nearly simultaneous peaks in both the high-γ activity and MUA; specifically within a window defined by the midpoint of the IDI preceding and following each discharge. Over all seizures in which the MEA was recruited into the ictal core, 1.7% of discharges were rejected due to this criterion (12 peak detections out of 708 peak detections, recorded from six seizures). Per-discharge correlations among firing rate and high-γ measures were evaluated with Spearman's *ρ*. IDIs were calculated from the mean firing rate and high-γ amplitude over all recorded MEA channels by subtracting each successive time at which peak firing rate or high-γ occurred.

We used the discharge width to quantify the temporal dispersion of multiunit firing. Discharge width was measured by first converting multiunit timestamps into *N* × *M* binary matrices, where *N* represented the number of channels on the array and *M* represented the number of milliseconds from seizure start through termination. Each element of the matrix was either one or zero, representing the presence or absence of an action potential on that specific channel during that specific millisecond. The sum of this matrix over the channel dimension for each discharge, as defined by the aforementioned criteria, was fit to a Gaussian and the s.d. of the fit was operationally defined as the discharge width (for example, black dotted lines in Fig. 7a). Discharges with *R*^2^ values <0.9 were excluded from this analysis (9.2% of discharges, or 64 of 696 discharges).

Mutual information was used as a measure of multiunit timing coincidence^68^. Mutual information for action potential timing was measured by taking 100-bit binary words from each channel in the aforementioned action potential matrix. Mutual information was then calculated for these 100-bit words every 25 ms through the duration of the seizure for each pairwise combination of channels across the MEA. The discharge amplitude was defined as the peak median mutual information across combinations of channels normalized by the firing rate at that point.

## Additional information

**How to cite this article:** Smith, E. H. *et al*. The ictal wavefront is the spatiotemporal source of discharges during spontaneous human seizures. *Nat. Commun.* 7:11098 doi: 10.1038/ncomms11098 (2016).

## Supplementary Material

Supplementary Information Supplementary Figures 1-6, Supplementary Tables 1-2, Supplementary Notes 1-2 and Supplementary References

Supplementary Movie 1 Traveling wave direction is dependent on the ictal wavefront. The movie steps through discharges from pre-recruitment to post-recruitment. The gray trace along the top of the frame shows the low frequency LFP. The current discharge appears color coded as in Figure 6a. The lower left map shows the ranked maximally decreasing slopes across the array for the current discharge with lighter-copper bursts occurring later, as in Figures 5 and S3. The lower right compass plot shows the direction of the ictal wavefront in the thick black line, and the direction of traveling waves color-coded as in Figure 6a.
