# Supplementary material for: The ictal wavefront is the spatiotemporal source of discharges during spontaneous human seizures
Source: Nat Commun. 2016 Mar 29;7:11098. doi: 10.1038/ncomms11098 (PMC4820627; doi:10.1038/ncomms11098)
Supplement: Supplementary Information — Supplementary Figures 1-6, Supplementary Tables 1-2, Supplementary Notes 1-2 and Supplementary References [file ncomms11098-s1.pdf]

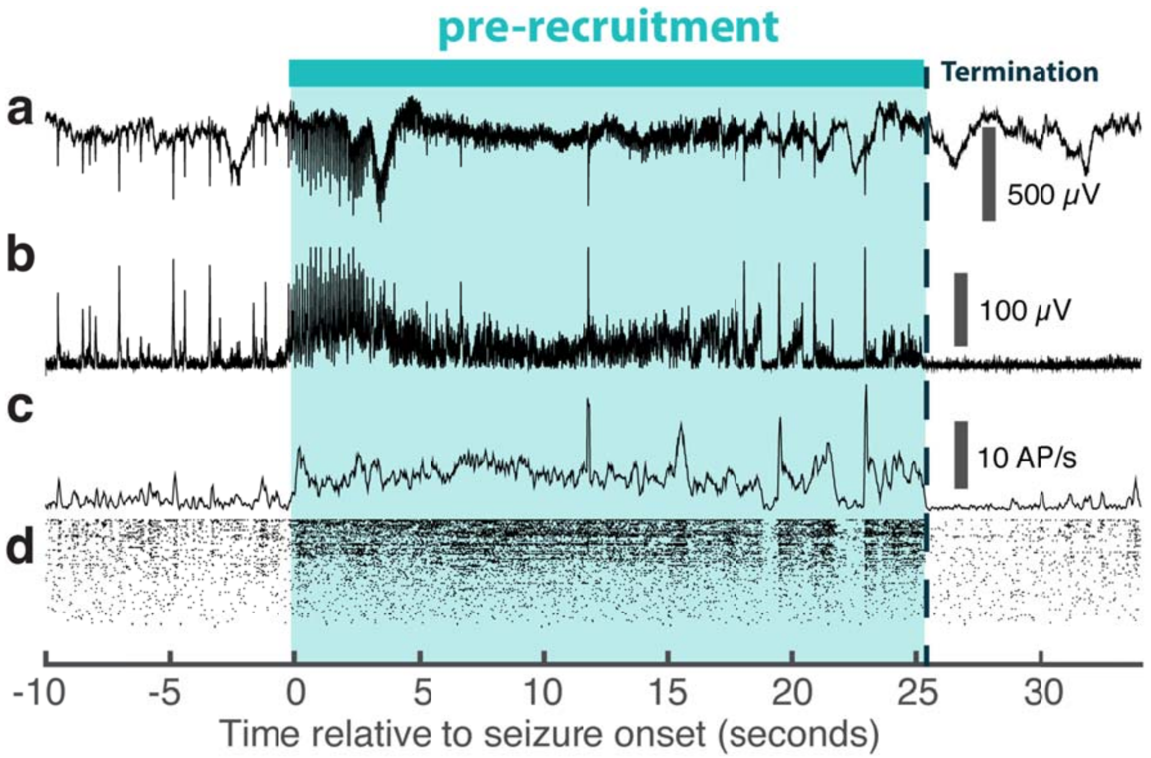

Supplementary Figure 1. Progression of seizure activity recorded from a microelectrode array that was not recruited into the ictal core. (**a**) Raw LFP traces recorded from a single microelectrode during a seizure. (**b**) Averaged high-gamma over electrodes on the array during the seizure in **a**. (**b**) Averaged multiunit firing rate over electrodes on the array during the seizure in **a**. (**d**) Multiunit raster plot over microelectrode array channels during the seizure in **a**.

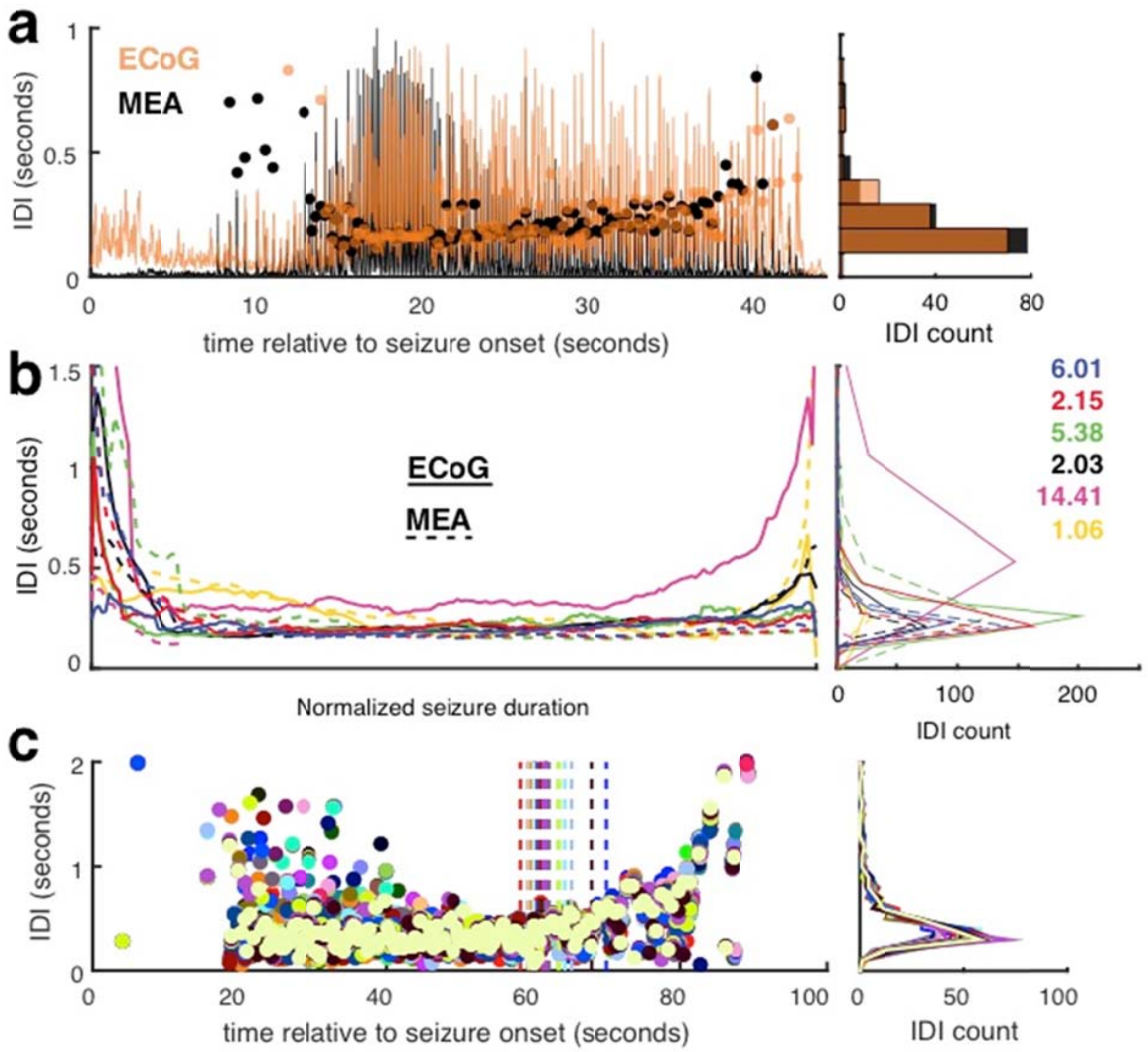

Supplementary Figure 2. Inter-discharge intervals are consistent across electrophysiological scales and are related to seizure stage. (**a**) Inter-discharge intervals (IDI) for a seizure recorded from patient 5 with the MEA (black circles), and an adjacent ECoG electrode (orange circles). Ictal high gamma traces are shown in corresponding colors for each recording modality. An IDI histogram is shown to the right of the IDI plots through time. (**b**) IDIs for all ictal core seizures (different colors) recorded on the MEA (dotted lines) and nearest ECoG electrodes (solid lines). IDI histograms are again shown to the right as dotted (MEA) and solid lines (ECoG). The Kullback-Liebler (K-L) divergences between distributions of IDIs recorded on the MEA and ECoG are shown above these histograms using the corresponding color for each seizure. The seizure in which the MEA recording was curtailed is shown in pink and accordingly has a higher K-L divergence. (**c**) IDIs determined from each ECoG electrode (different colors) during a single seizure, with IDI histograms again shown to the right. The dotted lines for each correspondingly colored ECoG electrode indicate where the division between post-recruitment and pre-termination epochs occurred, based on the coefficient of variation of the IDIs.

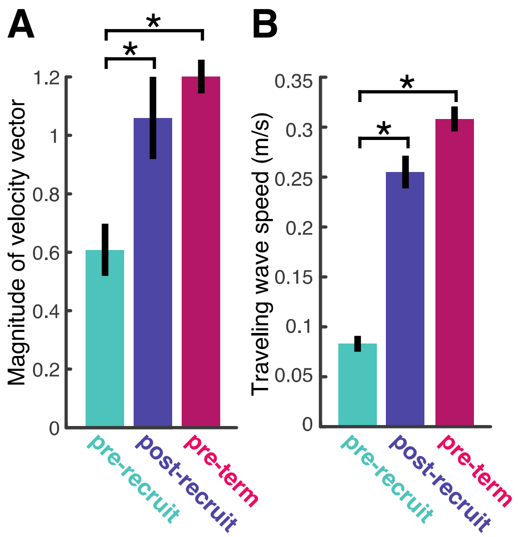

Supplementary Figure 3. Alternative measures of traveling wave speed across the electrode array.

(**a**) Mean magnitude of the velocity vector quantified over six recruited seizures in three epochs (color-coded as in Figure 1). (**b**) Mean traveling wave speed quantified over six recruited seizures in three epochs (color-coded as in Figure 1). Asterisks indicate significant comparisons. Bars indicate standard error.

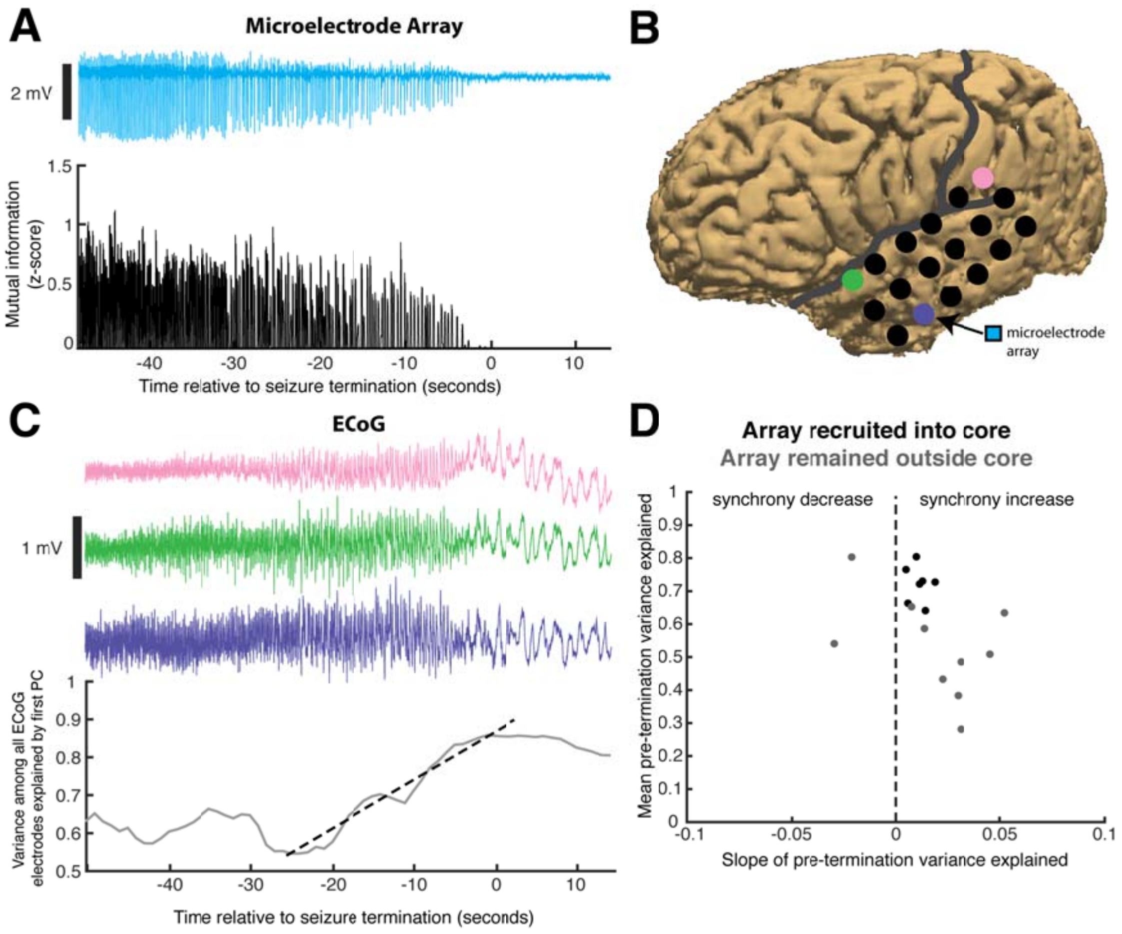

Supplementary Figure 4. Large-scale pre-termination increases in LFP synchrony. (**a**) The end of a seizure recorded on the microelectrode array is shown in blue. Median mutual information for multiunit event timing among pairs of microelectrodes is shown below for the same seizure. (**b**) Reconstruction of ECoG and microelectrode locations for the patient from whom seizures in A and C were recorded. Colored electrodes correspond to voltage traces in **a** and **c.** (**c**) Three channels of LFP recorded from ECoG electrodes during the same seizure as in A and a representative increase in variance explained by the first principal component, across all ECoG electrodes on the grid shown in **b,** during the pre-termination epoch. The black dotted line shows linear regression to determine the slope of the variance explained during the pre-termination epoch for the example seizure. (**d**) Scatter plot of pre-termination increases in variance, and mean amount of variance explained for each seizure in patients with microelectrodes implanted in the ictal core (black) and out of the ictal core (gray).

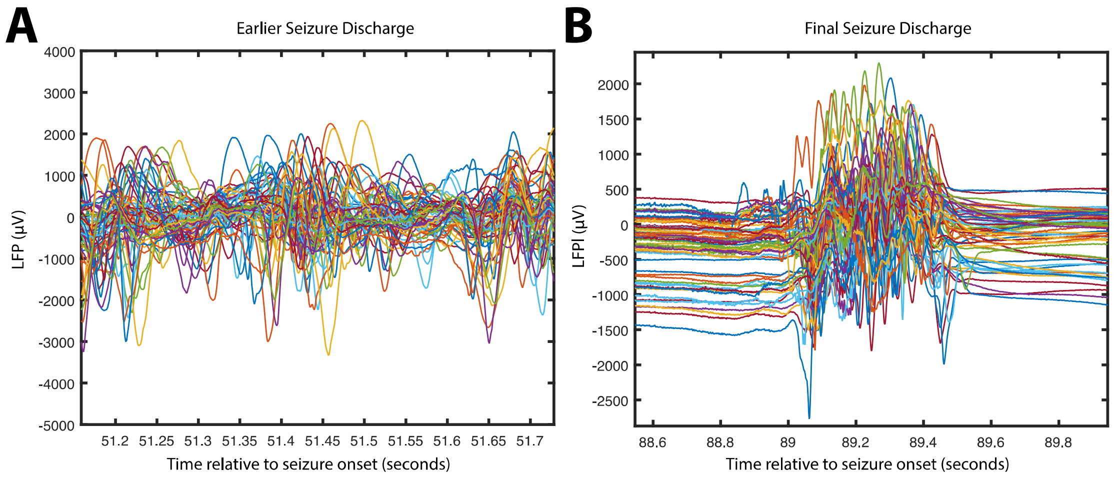

Supplementary Figure 5. Illusory hypersynchrony in low frequency LFP. (**a**) Low frequency LFP recorded from grid ECoG electrodes during a discharge that occurred early in the pre-termination phase of the seizure. Each colored trace represents a different electrode. (**b**) Low frequency LFP recorded from grid ECoG electrodes during the final discharge in the seizure. Each colored trace represents a different electrode.

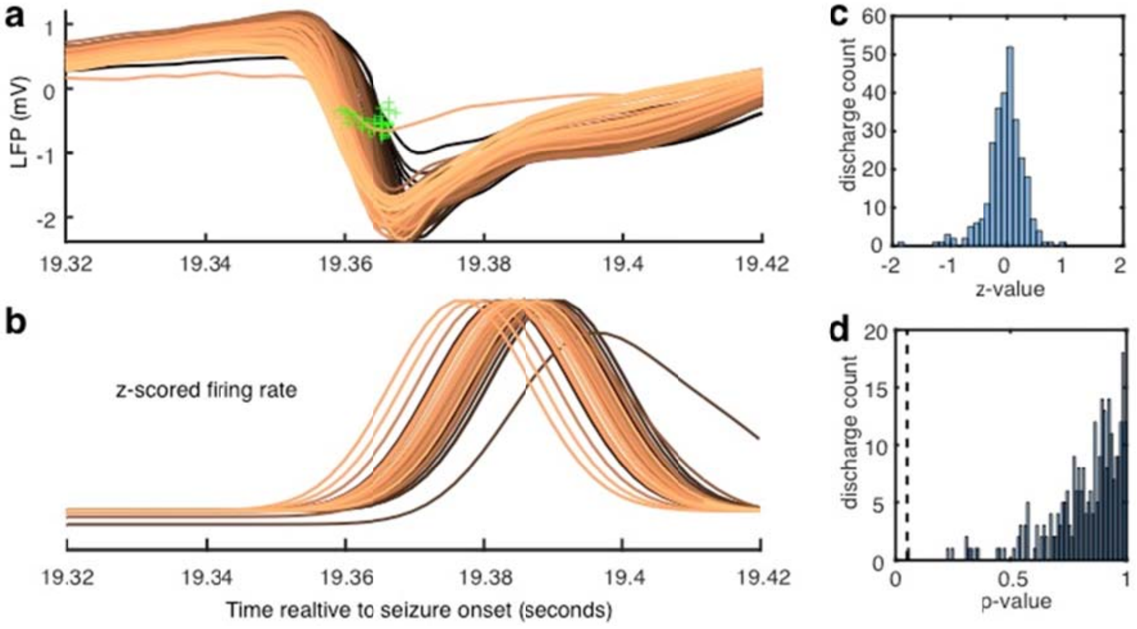

Supplementary Figure 6. Traveling waves across MEA microelectrodes and corresponding multiunit activity delays. (**a**) Example LFP discharges recorded across the MEA, colored by when their points of maximum descent (green crosses) occur. Colors range from copper (earlier) to black (later). (**b**) Normalized firing rate on each channel in **a**. Matching colors indicate the signals were recorded on the same MEA channels. (**c**) Histogram of z-values for Wilcoxon signed-rank tests between MEA channel ranks determined from traveling wave slopes and multiunit activity peaks across all pre-termination discharges from all seizures. (**d**) Histogram of p-values for Wilcoxon signed-rank tests between MEA channel ranks determined from traveling wave slopes and multiunit activity peaks across all pre-termination discharges from all seizures. The dotted-line indicates a critical value of 0.05. All of the p-values were greater than 0.05.

Supplementary Table 1. Computational model parameters

Parameter	Notation	Value
**Input resistance**	$R_{input} = 1/g_{rest}$	125 MΩ
**Resting membrane potential**	E_{rest}	-68 mV
**Mean maximal intra-network synaptic conductance**	g_{syn}	60 nS
**Mean intra-network synaptic conductance reversal potential**	E_{syn}	-30 mV
**Extra-network feedforward input**	g_{input}	0 ~ 3 nS
**Reversal potential of extra-network feedforward input**	E_{input}	0 mV
**Synaptic depression constant**	τ_D	200 ms
**Synaptic depression ratio**	f_D	0.6
**Mean maximal firing rate**	r_0	100 Hz
**Spiking threshold**	V_{th}	-42 mV
**Spiking threshold standard deviation**	σ	4.5 mV
**Synaptic time constant**	τ_v	25 ms

Supplementary Table 2. Clinical details from study patients

**Patient (Age/Gender)**	**1 (30/F)**	**2 (30/M)**	**3 (32/F)**	**4 (19/F)**	**5 (24/M)**
**Array Recruited into ictal Core?**	No (penumbral)	No (penumbral)	Yes	Yes	Yes
**MEA location**	Left supplementary motor area, 3 cm superior to Broca's area	Left lateral frontal, 2 cm superior to Broca's area	Left inferior temporal gyrus, 2.5 centimeters from temporal pole	Right posterior temporal, 1cm inferior to angular gyrus	Left middle temporal gyrus 3 cm from temporal pole
**Clinically – Defined Seizure Onset Zone**	Left supplementary motor area (including MEA site)	Left frontal operculum (including MEA site)	Left basal/anterior temporal (including MEA site)	Right posterior lateral temporal (including MEA site)	Left mesial temporal lobe spread to lateral temporal lobe (including MEA site)
**Number of Seizures examined**	7	3	3	1 (MEA recording curtailed during post-recruitment epoch)	2
**Seizure Types**	Complex partial	Complex partial	Complex partial	Complex partial with secondary generalization	Complex partial
**Pathology**	N/A (multiple subpial transections performed)	Nonspecific	Mild CA1 Neuronal loss; lateral temporal nonspecific	Nonspecific	Nonspecific (no hippocampal sclerosis)
**Outcome**	Engel III	Engel 1a	Engel 1a	Engel 1a	Engel 1a

Supplementary Note 1

LFP synchrony increases toward the end of seizures

We observed decreased MUA desynchronization over the MEA (Supplementary Figure 4A), and corresponding decreases in high gamma over ictal core ECoG sites in all five patients leading up to seizure termination. We interpreted these signals to be indicative of a progressive desynchronization leading up to seizure termination, which is in contrast to the increased synchrony observed in previous ECoG studies [1-4]. In order to confirm large-scale (centimeters of brain area) pre-termination synchrony in the current dataset, and control for the possibility that the seizures examined here have different dynamics than those reported in the literature, we examined synchrony in the broadband LFP recorded across the ECoG grids overlying each MEA channel. Synchrony was measured in a similar manner to previous studies [1,5], by examining the amount of variance that can be explained by the single largest covariance dimension using principal components analysis. We found that synchrony increased across the ECoG grids prior to seizure termination, consistent with previous observations. However, this increase in synchrony was apparent on ECoG electrodes in patients with the MEA in either the ictal core or the penumbra (Supplementary Figure 4B and 4C). There was also no significant difference among patients in the pre-termination increase in synchrony (Mann-Whitney U, $p = 0.12$, Supplementary Figure 4D). These results suggest that previously observed increases in synchrony are dominated by the low frequency

LFP, which accounts for an exponentially large portion of the ECoG signal variance, compared to high gamma.

Supplementary Note 2

Computational Model

We modeled macrocolumns of cells in the ictal core using a mean field approach, where the firing rate of a typical cell was used to approximate the entire macrocolumn. Cells received intra-network recurrent synaptic input and extra-network current input. The typical cell membrane potential, V, was modeled with the following set of equations:

$$\tau_v \frac{dV}{dt} = V_\infty - V$$

$$V_\infty = \frac{g_{rest} * E_{rest} + g_{syn} * \mathrm{D} * \mathrm{f(V)} * E_{syn} + g_{input} * E_{input}}{g_{rest} + g_{syn} * \mathrm{D} * \mathrm{f(V)} + g_{input}}$$

$$\tau_v = \frac{C}{g_{rest} + g_{syn} * \mathrm{D} * \mathrm{f(V)} + g_{input}}$$

Where τ_v is the effective time constant for change in the membrane potential over time and V_∞ is the steady state membrane potential. Input to the network of cells was represented with the g_{input} term and recurrent connections among cells were

treated as intra-network synaptic input, which was modeled with the $g_{syn} * \mathrm{D} *$ $\mathrm{f(V)} * E_{syn}$ term. In this term, g_{syn} and f(V) stand for maximal intra-network recurrent synaptic conductance and firing probability as a function of membrane potential, respectively. Firing probability followed the following error function

$$f(V) = \int_{-\infty}^{V} \frac{1}{\sqrt{2\pi}\sigma} e^{-(u-V_{th})^2/2\sigma^2} du$$

Synaptic depression evoked by repeated activations was included in the model. Because repetitive synaptic transmissions in the cerebral cortex has been found to induce short-term synaptic depression (STD)[6], the effective synaptic strength D was modeled with the following set of equations:

$$\tau_D \frac{dD}{dt} = D_\infty - D$$

$$D_\infty = \frac{1}{1 + (1 - f_D) * r_0 * f(V) * \tau_D}$$

Where $f_D$represents the synaptic depression ratio (i.e how much a post synaptic potential is depressed following a preceding potential) and r_0 represents a typical cell's maximal firing rate. D_∞ was obtained by assuming a pre-synaptic Poisson spiking property [7]. Note that very sophisticated, biologically inspired models exhibit similar properties [8].

Table S1 shows parameters used for computational simulation. The parameters were chosen based on experimental data from layer 5 pyramidal neurons [9,10]. Two exceptions were the mean maximal recurrent conductance and average reversal potential, g_{syn} and E_{syn} respectively. The value of E_{syn} was chosen by averaging the reversal potentials of GABA and glutamate receptors in order to model both inhibitory and excitatory intra-network connections. The value of g_{syn} was chosen to make the typical cells demonstrate tonic firing while receiving maximal recurrent input. Numerical results were calculated by XPP with a 0.1 ms time step using the Runge–Kutta fourth order method [11].

The computational model was thus established, and decreasing input was applied in order to simulate the effects of a progressively weakening or moving ictal wavefront. The value of g_{input} was therefore decreased linearly from 3 to 0 nS, with seizure dynamics being reproduced between approximately 2.7 and 2.2 nS.

Cortical reconstruction and ECoG alignment

Cortical reconstruction and volumetric segmentation was performed with the Freesurfer image analysis suite, which is documented and freely available for download online (http://surfer.nmr.mgh.harvard.edu/).

Supplementary References

1. Schindler, K., Elger, C. E. & Lehnertz, K. Increasing synchronization may promote seizure termination: evidence from status epilepticus. *Clin Neurophysiol* **118,** 1955–1968 (2007).
2. Kramer, M. A. & Cash, S. S. Epilepsy as a disorder of cortical network

organization. *Neuroscientist* **18,** 360–372 (2012).
3. Kramer, M. A. *et al.* Coalescence and fragmentation of cortical networks during focal seizures. *Journal of Neuroscience* **30,** 10076–10085 (2010).
4. Jiruska, P. *et al.* Synchronization and desynchronization in epilepsy: controversies and hypotheses. *J Physiol (Lond)* **591,** 787–797 (2013).
5. Schindler, K., Leung, H., Elger, C. E. & Lehnertz, K. Assessing seizure dynamics by analysing the correlation structure of multichannel intracranial EEG. *Brain* **130,** 65–77 (2007).
6. Dobrunz, L. E. & Stevens, C. F. Heterogeneity of release probability, facilitation, and depletion at central synapses. *Neuron* **18,** 995–1008 (1997).
7. Dayan, P. & Abbott, L. F. Theoretical neuroscience. (2001).
8. Markram, H., Wang, Y. & Tsodyks, M. Differential signaling via the same axon of neocortical pyramidal neurons. *Proc Natl Acad Sci USA* **95,** 5323–5328 (1998).
9. Tripathy, S. J., Savitskaya, J., Burton, S. D., Urban, N. N. & Gerkin, R. C. NeuroElectro: a window to the world's neuron electrophysiology data. *Front Neuroinform* **8,** 40 (2014).
10. Williams, S. R. & Stuart, G. J. Site independence of EPSP time course is mediated by dendritic I(h) in neocortical pyramidal neurons. *J Neurophysiol* **83,** 3177–3182 (2000).
11. Ermentrout, B. Simulating, Analyzing, and Animating Dynamical Systems: A Guide to XPPAUT ... - Bard Ermentrout - Google Books. (2002).
